# Supplementary figures and images for: A SNP variation in the Sucrose synthase (SoSUS) gene associated with sugar-related traits in sugarcane
Source: PeerJ. 2023 Dec 15;11:e16667. doi: 10.7717/peerj.16667 (PMC10726748; doi:10.7717/peerj.16667)

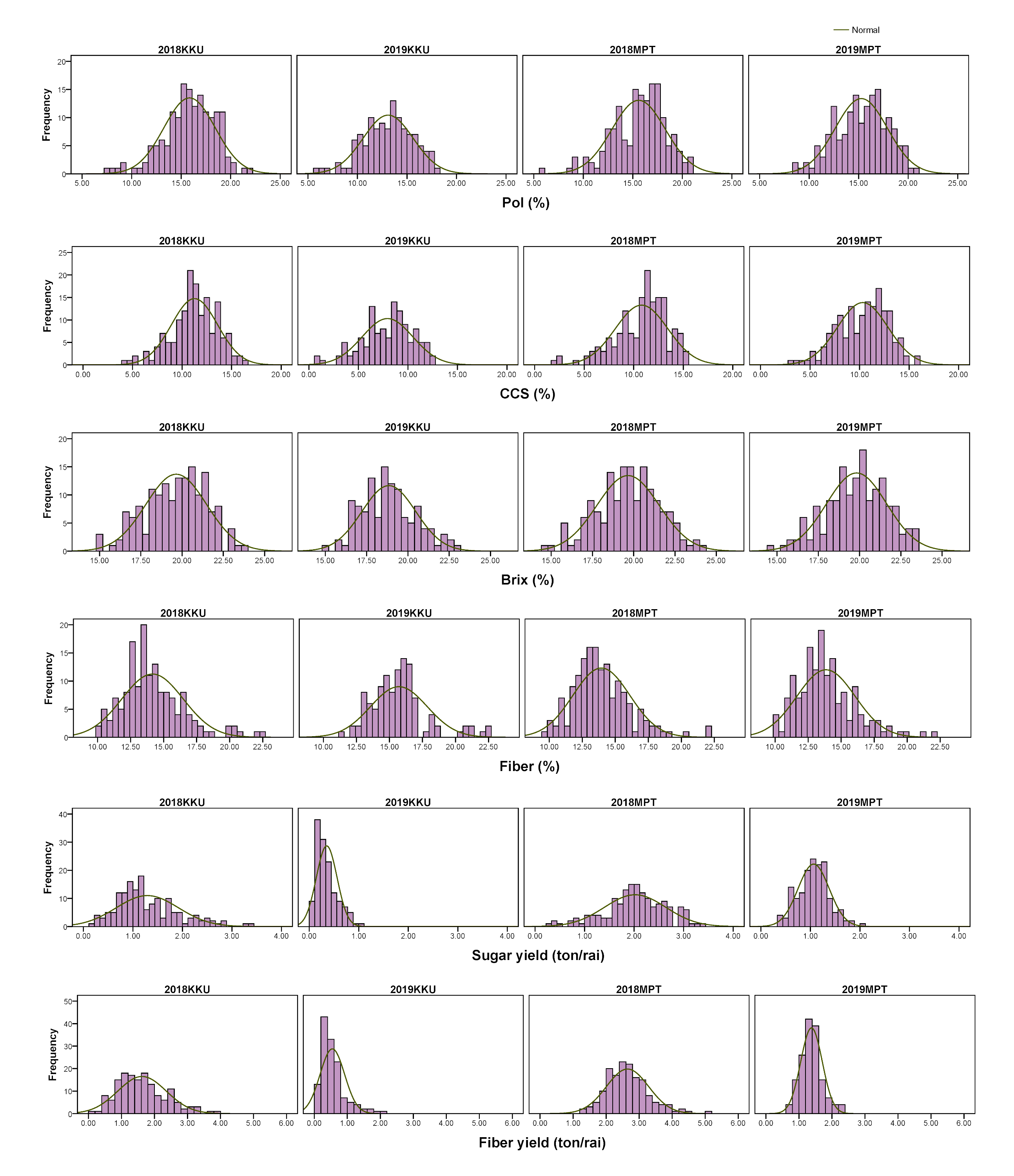

Supplement: Supplemental Information 1 — 2018, 2017–2018 cropping season (plant cane); 2019, 2018–2019 cropping season (first ratoon); KKU, Khon Kaen; MPT, Mitr Phol Innovation and Research Center. [file peerj-11-16667-s001.png]

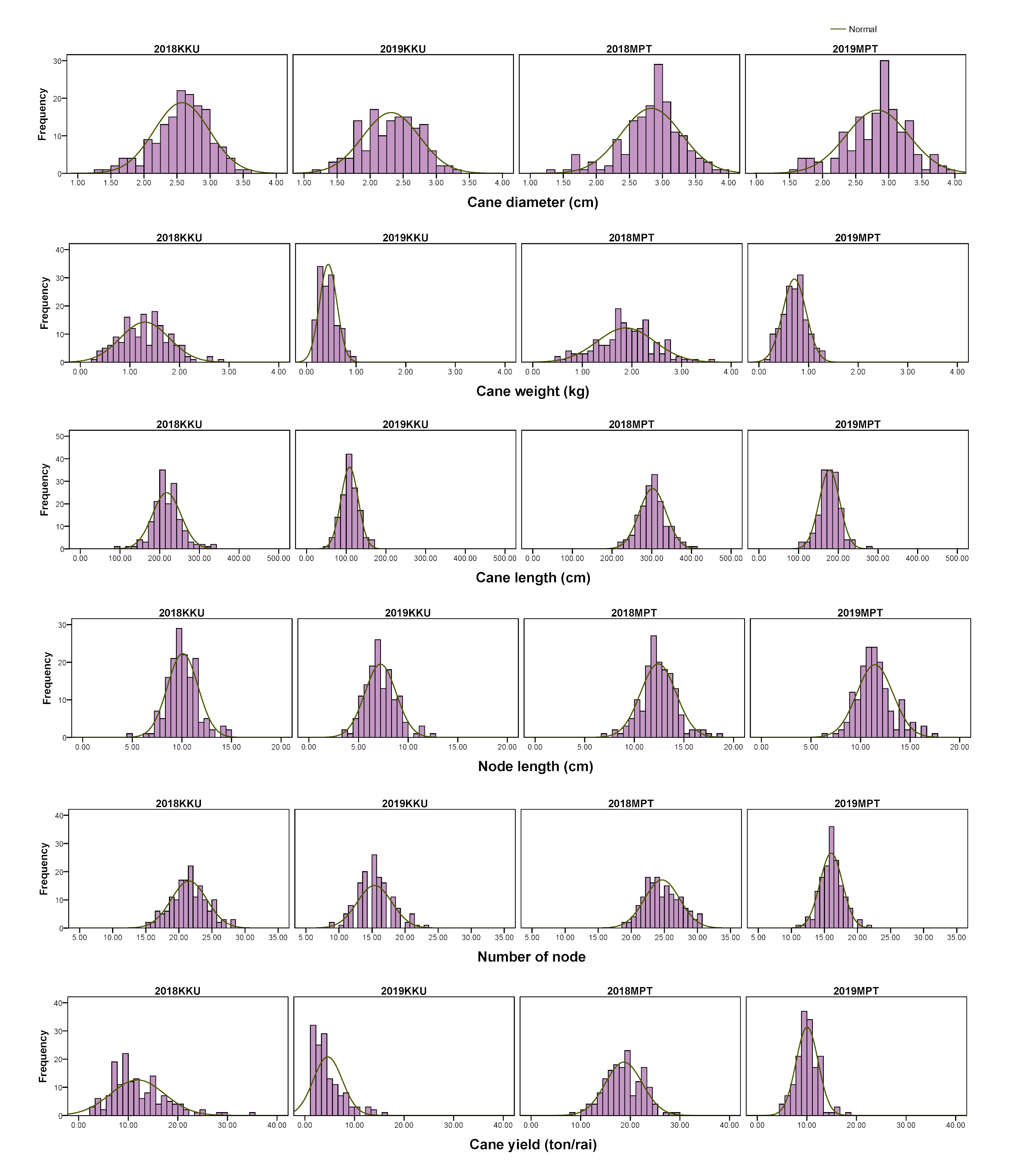

Supplement: Supplemental Information 2 — 2018, 2017–2018 cropping season (plant cane); 2019, 2018–2019 cropping season (first ratoon); KKU, Khon Kaen; MPT, Mitr Phol Innovation and Research Center. [file peerj-11-16667-s002.png]

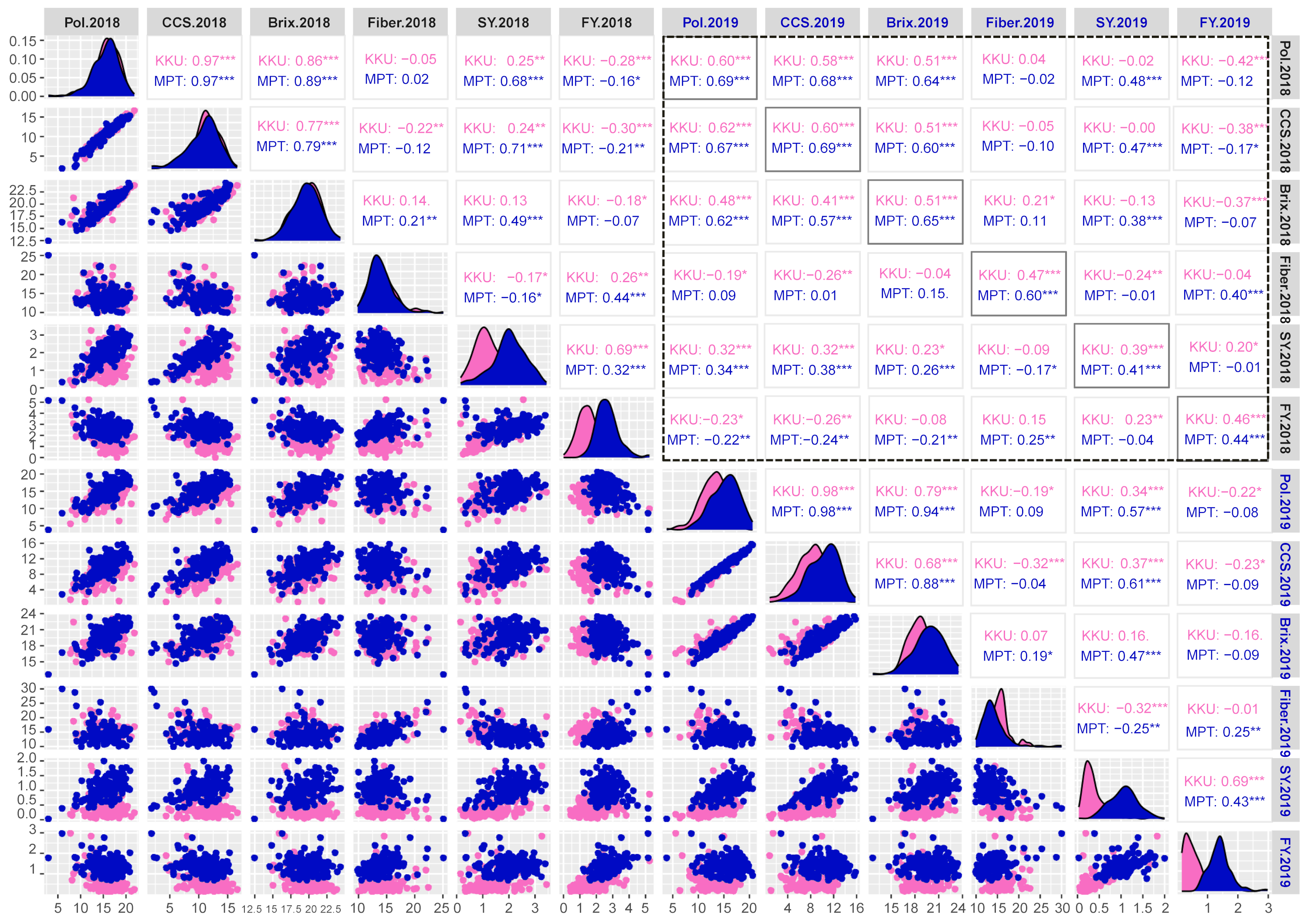

Supplement: Supplemental Information 3 — The lower triangular matrix is composed of scatter plots in which the pink dots represent the Khon Kaen (KKU) location, while the blue dots represent the Mitr Phol Innovation and Research Center (MPT) location. The upper triangular matrix shows Spearman correlation coefficients and significant difference for traits at both locations, KKU (pink) and MPT (blue). Black dashed-lined frame indicates the correlation coefficients between the traits among two years. The main diagonal comprises the density-based histogram plots of the traits at both locations, KKU (pink) and MPT (blue). SY, sugar yield; FY, fiber yield. 2018, 2017–2018 cropping season (plant cane); 2019, 2018–2019 cropping season (first ratoon). *p < 0.05; **p < 0.01; ***p < 0.001 [file peerj-11-16667-s003.png]

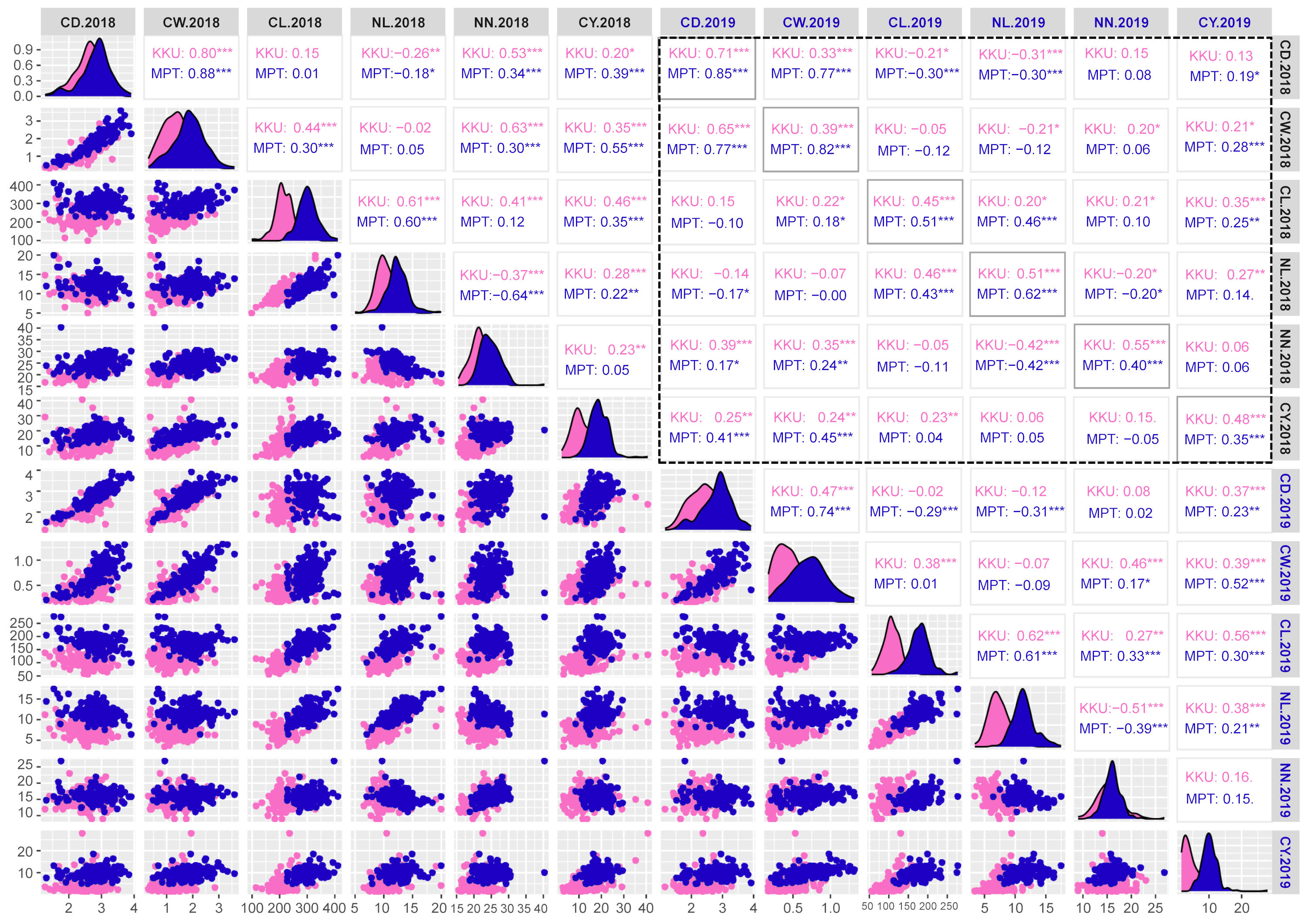

Supplement: Supplemental Information 4 — The lower triangular matrix is composed of scatter plots in which the pink dots represent the Khon Kaen (KKU) location, while the blue dots represent the Mitr Phol Innovation and Research Center (MPT) location. The upper triangular matrix shows Spearman correlation coefficients and significant difference for traits at both locations, KKU (pink) and MPT (blue). Black dashed-lined frame indicates the correlation coefficients between the traits among two years. The main diagonal comprises the density-based histogram plots of the traits at both locations, KKU (pink) and MPT (blue). CD, cane diameter; CW, cane weight; CL, cane length; NL, node length; NN, number of nodes; CY, cane yield. 2018, 2017–2018 cropping season (plant cane); 2019, 2018–2019 cropping season (first ratoon). *p < 0.05; **p < 0.01; ***p < 0.001. [file peerj-11-16667-s004.png]

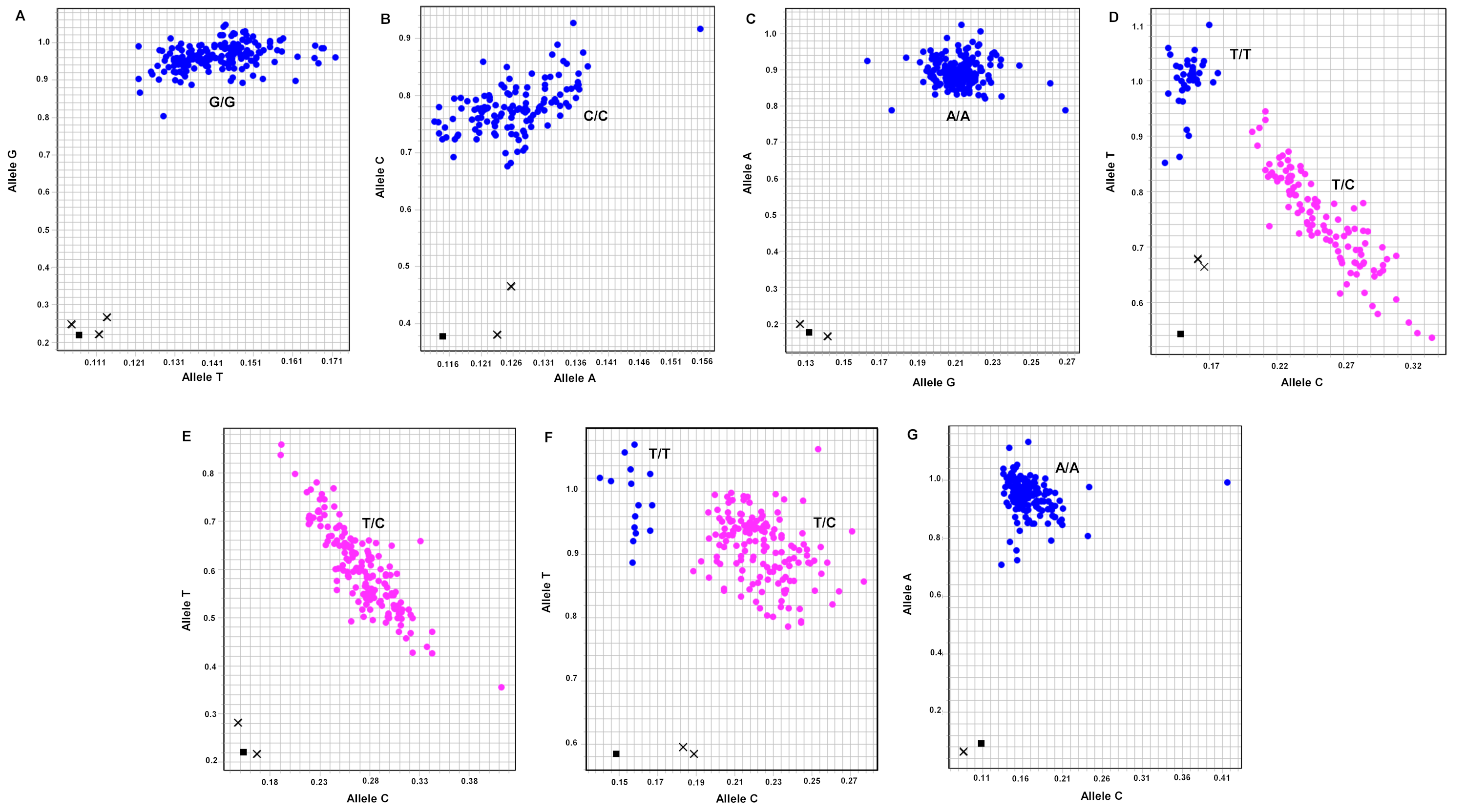

Supplement: Supplemental Information 5 — (A) mSoLSD1_SNPch.G/T (blue: genotypes G/G), (B) mSoLSD1_SNPch.C/A (blue: genotypes C/C), (C) mSoCALR_SNPch2.A/G (blue: genotypes A/A), (D) mSoSUS1_SNPCh10.T/C (blue: genotypes T/T and pink: T/C), (E) mSoRH56_SNPCh3.T/C (pink: genotypes T/C), (F) mSoKAN1_SNPCh7.T/C(blue: genotypes T/T and pink: T/C), and (G) mSoNHX7_SNPCh8.A/C (blue: genotypes A/A). Blue dots represent the homozygous genotypes and the pink dots represent heterozygous genotypes. [file peerj-11-16667-s005.png]
